# Neonatal Encephalopathy With Group B Streptococcal Disease Worldwide: Systematic Review, Investigator Group Datasets, and Meta-analysis

**DOI:** 10.1093/cid/cix662

**Published:** 2017-11-06

**Authors:** Cally J Tann, Kathryn A Martinello, Samantha Sadoo, Joy E Lawn, Anna C Seale, Maira Vega-Poblete, Neal J Russell, Carol J Baker, Linda Bartlett, Clare Cutland, Michael G Gravett, Margaret Ip, Kirsty Le Doare, Shabir A Madhi, Craig E Rubens, Samir K Saha, Stephanie Schrag, Ajoke Sobanjo-ter Meulen, Johan Vekemans, Paul T Heath, for the GBS Neonatal Encephalopathy Investigator Group, Alfredo Garcia-Alix, Nem-Yun Boo, Miriam Martinez-Biarge, Jeanie Cheong, Frances Cowan, Linda S de Vries, Gemma Arca-Diaz, A David Edwards, Matthew Ellis, Christopher Gale, Hannah C Glass, Floris Groenendaal, Alistair Gunn, Breda Hayes, Susan E Jacobs, Clark T Johnson, Gugu Kali, Manogna Manne, An N Massaro, Nicola J Robertson, Prakeshkumar Shah, Seetha Shankaran, Cally J Tann, Sudhin Thayyil, Marianne Thoresen, Brian H Walsh, Pia Wintermark, Anne C C Lee

**Affiliations:** 1 Maternal, Adolescent, Reproductive and Child Health Centre, London School of Hygiene & Tropical Medicine, United Kingdom;; 2 Neonatal Medicine, University College London Hospitals NHS Foundation Trust, United Kingdom;; 3 Institute for Women’s Health, University College London, United Kingdom;; 4 College of Health and Medical Sciences, Haramaya University, Dire Dawa, Ethiopia;; 5 Medical School, University College London, United Kingdom;; 6 King’s College London, United Kingdom;; 7 Departments of Pediatrics and Molecular Virology and Microbiology, Baylor College of Medicine, Houston, Texas;; 8 Department of International Health, Johns Hopkins Bloomberg School of Public Health, Baltimore, Maryland;; 9 Medical Research Council: Respiratory and Meningeal Pathogens Research Unit, and Department of Science and Technology/National Research Foundation: Vaccine Preventable Diseases, Faculty of Health Sciences, University of the Witwatersrand, Johannesburg, South Africa;; 10 Global Alliance to Prevent Prematurity and Stillbirth, Seattle, Washington;; 11 Department of Obstetrics and Gynecology, University of Washington, Seattle;; 12 Department of Microbiology, Faculty of Medicine, Chinese University of Hong Kong;; 13 Centre for International Child Health, Imperial College London, United Kingdom;; 14 Vaccine Institute, Institute for Infection and Immunity, St George’s Hospital, University of London and St George’s University Hospitals NHS Foundation Trust, United Kingdom;; 15 National Institute for Communicable Diseases, National Health Laboratory Service, Johannesburg, South Africa;; 16 Department of Global Health, University of Washington, Seattle;; 17 Bangladesh Institute of Child Health, Dhaka;; 18 National Center for Immunization and Respiratory Diseases, Centers for Disease Control and Prevention, Atlanta, Georgia;; 19 Bill & Melinda Gates Foundation, Seattle, Washington; and; 20 World Health Organization, Geneva, Switzerland

**Keywords:** group B *Streptococcus*, newborn, neonatal encephalopathy, hypoxic-ischemic encephalopathy, therapeutic hypothermia

## Abstract

**Background:**

Neonatal encephalopathy (NE) is a leading cause of child mortality and longer-term impairment. Infection can sensitize the newborn brain to injury; however, the role of group B streptococcal (GBS) disease has not been reviewed. This paper is the ninth in an 11-article series estimating the burden of GBS disease; here we aim to assess the proportion of GBS in NE cases.

**Methods:**

We conducted systematic literature reviews (PubMed/Medline, Embase, Latin American and Caribbean Health Sciences Literature [LILACS], World Health Organization Library Information System [WHOLIS], and Scopus) and sought unpublished data from investigator groups reporting GBS-associated NE. Meta-analyses estimated the proportion of GBS disease in NE and mortality risk. UK population-level data estimated the incidence of GBS-associated NE.

**Results:**

Four published and 25 unpublished datasets were identified from 13 countries (N = 10436). The proportion of NE associated with GBS was 0.58% (95% confidence interval [CI], 0.18%–.98%). Mortality was significantly increased in GBS-associated NE vs NE alone (risk ratio, 2.07 [95% CI, 1.47–2.91]). This equates to a UK incidence of GBS-associated NE of 0.019 per 1000 live births.

**Conclusions:**

The consistent increased proportion of GBS disease in NE and significant increased risk of mortality provides evidence that GBS infection contributes to NE. Increased information regarding this and other organisms is important to inform interventions, especially in low- and middle-resource contexts.

Intrapartum complications with hypoxic brain injury is one of the leading causes of neonatal mortality and long-term impairment morbidity worldwide [1]. Newborns exposed to a perinatal insult typically present with neonatal encephalopathy (NE), a descriptive term for a clinical constellation of neurological dysfunctions in the term infant [2]. Many cases of NE, and the often-resultant neonatal death or stillbirth, are likely to result from a complex multifactorial pathway to brain injury to which hypoxia-ischemia substantially contributes [3–6].

Hypoxic-ischemic encephalopathy (HIE) is a term used to define those cases with NE likely due to hypoxia-ischemia. Across many high-income countries (HICs), it is now standard of care for infants with moderate to severe HIE to be treated with whole-body therapeutic hypothermia or “cooling,” where their core body temperature is cooled to 33.5°C for 72 hours, followed by a controlled period of rewarming [7, 8]. Systematic reviews of cooling trials have shown that therapeutic hypothermia is able to reduce the combined outcome of death or major neurodevelopmental disability in survivors, with a number needed to treat = 7 for an additional beneficial outcome [9]. To define which infants should receive cooling, clinical criteria have been developed to identify NE assumed to be due to hypoxia-ischemia. The presence of other comorbidities such as acute perinatal infection, however, does not usually preclude cooling treatment. A systematic review of cooling in low- and middle-income countries (LMICs) failed to show a statistically significant reduction in neonatal mortality [10]; however, individual centers in middle-income settings have reported favorable outcomes [11].

Increasing evidence suggests the importance of a sensitizing effect of inflammation, increasing the susceptibility of the immature brain to perinatal events that drive the pathogenesis of NE [12, 13]. Animal studies have shown that exposure to bacterial endotoxin increases vulnerability of the developing brain to hypoxia-ischemia [14–16]. Other study findings have shown a temporal relationship suggesting that exposures can be sensitizing or preconditioning to the fetal and neonatal brain [13, 15, 16]. A recent study, modeling gram-positive infection prior to hypoxic-ischemic injury, demonstrated sensitization of the brain to injury, but also encouragingly, neuroprotection with hypothermia [17]. In clinical studies, factors associated with intrauterine infection and inflammation, such as prolonged rupture of membranes, have been shown to be associated with NE [5], and the presence of placental inflammation/infection has been shown to be independently associated with an increased risk of NE in both high- and low-income settings [18, 19]. While few clinical studies have examined the role of specific infections and inflammation as independent risk factors for NE, an important role is hypothesized [20, 21].

Group B *Streptococcus* (GBS; *Streptococcus agalactiae*) is an important pathogen for newborns and is one of the most common causes of neonatal infection worldwide [22]. Defining the contribution of GBS to other important and common neonatal conditions, such as NE, is important to fully understand the global burden of GBS in pregnant women and infants, and because it may become potentially preventable, through maternal GBS vaccination.

This article aims to examine the proportion with GBS disease among infants with NE (Figure 1).

**Figure 1.**
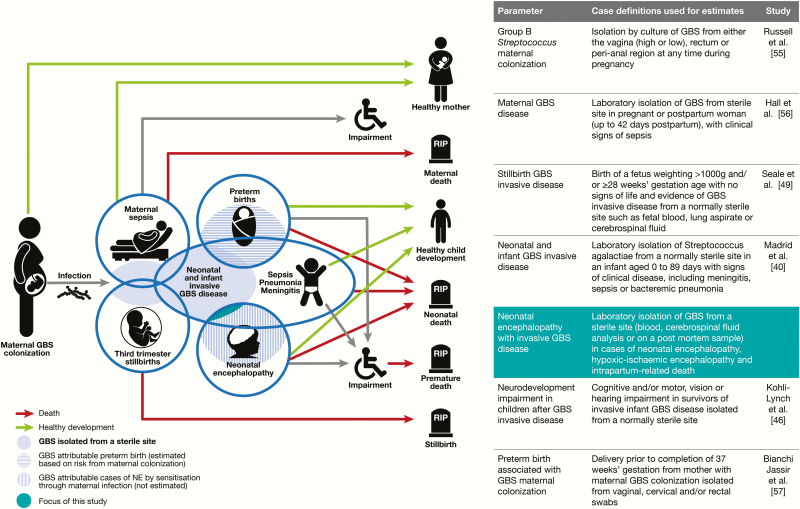
Neonatal encephalopathy (NE) is part of the compartmental model to estimate the burden of group B streptococcal (GBS) disease, as described by Lawn et al [54].

## Objectives

1. To undertake to provide a comprehensive and systematic literature review and identify unpublished cohorts through an investigator group formed through trials and registries of infants with NE assumed to be due to hypoxia-ischemia meeting criteria for therapeutic hypothermia, in order to analyze the risk of invasive GBS disease among neonates with NE.
2. To assess the data available for the proportion with invasive GBS disease among infants with NE in order to estimate the burden of GBS in pregnant women, stillbirths, and infants.
3. To evaluate data gaps and recommend improvements for data acquisition on NE associated with GBS disease.

## METHODS

This article is part of a protocol entitled “Systematic estimates of the global burden of GBS in pregnant women, stillbirths and infants,” submitted for ethical approval to the London School of Hygiene & Tropical Medicine (reference number 11966) and approved on 30 November 2016.

### Definitions

Case definitions used in this article include NE, HIE, intrapartum-related death, and birth asphyxia; these are shown in Supplementary Table 1, in addition to definitions of GBS sepsis, meningitis, and pneumonia. For the study of the association between GBS and NE, a case of GBS was defined as isolation of GBS, on either blood culture or molecular assay, from a normally sterile site (blood, cerebrospinal fluid, postmortem site).

## DATA SEARCHES AND INPUTS

### Published Data

We conducted systematic literature searches of Medline and Embase on 28 September 2016, and Literature in the Health Sciences in Latin America and the Caribbean (LILACS), the World Health Organization Library Information System (WHOLIS), and Scopus on 12 February 2017, and updated these on 21 March 2017. We searched with variants of terms related to “GBS,” “sepsis,” “asphyxia,” therapeutic hypothermia,” and “NE” (Supplementary Materials). Medical subject heading (MeSH) terms were used where possible. Supplementary Table 2 describes the full list of search terms. One investigator performed the database search, and screened for duplicates and for eligibility (M. V.). Two independent investigators (M. V. and N. R.) screened abstracts to assess their suitability for inclusion, and both reviewers subsequently extracted data. Where there was discrepancy between the 2 reviewers, a third investigator (C. T.) made the final decision. Additional articles were identified from reference lists through snowball searching. We did not apply date or language restrictions and texts were translated to English when published in other languages.

### Secondary Analysis of Data From Published Neonatal Encephalopathy Cohorts and Trials From the Investigator Group

Through snowball searching of reference lists, and a search on PubMed using terms related to infant/newborn/NE and therapeutic hypothermia (S.S.), cooling cohorts and trials were identified. As these articles did not include data related to GBS, the corresponding authors were contacted on a minimum of 2 occasions (at least 4 weeks apart) to inquire whether data on the proportion of GBS disease among infants with NE/HIE were available for secondary analysis. A consistent definition of GBS disease was used (Supplementary Table 1). Responding authors were requested to complete a standardized data collection spreadsheet summarizing the data.

### Unpublished Data From the Investigator Group

Unpublished data from patient registers in HICs were sourced from registers of therapeutic hypothermia (“cooling” registers), patient registers from neonatal neurology centers, and national neonatal research databases.

Neonatal neurology and cooling cohorts and registers were identified through literature review as above and through professional networking. Lead clinicians at the relevant sites were contacted and requested to complete the same standardized data collection spreadsheet summarizing the data to those providing data for secondary analysis above.

Centers known to be holding national neonatal data (United Kingdom, Canada, Australia, Norway) were contacted and invited to contribute data. Of these, the United Kingdom National Neonatal Research Database (NNRD) and the Canadian Neonatal Network (CNN) agreed to contribute data. The NNRD holds electronic patient record data, recorded by health professionals as part of routine clinical care, from United Kingdom neonatal units. Data held in the NNRD are cleaned; records with implausible data configurations are queried and corrected by the treating clinicians. The NNRD holds individual patient-level data from all admissions to National Health Service (NHS) neonatal units in England and Wales from 2012 and from all admissions to Scottish neonatal units from 2014. A formal comparison of NNRD data items against those recorded in case record forms of a multicenter, randomized placebo-controlled trial (Probiotics in Preterms [23]) demonstrated a high degree of data agreement (>95%) between the NNRD and clinical trial case report forms. The National Neonatal Research Database is a Clinical Dataset (National Neonatal Data Set) within the NHS Data Dictionary. Details of all data items are searchable at the following webpage: http://www.datadictionary.nhs.uk/data_dictionary/messages/clinical_data_sets/data_sets/national_neonatal_data_set/national_neonatal_data_set_-_episodic_and_daily_care_fr.asp?shownav=1.

For the purposes of this study, NE meeting the criteria for therapeutic hypothermia was defined as an infant >35 weeks’ gestation receiving at least 2 days of therapeutic hypothermia. The data were limited to infants receiving a minimum of 2 days’ therapeutic hypothermia to ensure exclusion of babies initially cooled but then rewarmed within a few hours after transfer to their tertiary cooling center and not found to adequately meet cooling criteria.

Data from Canada were extracted from the CNN database. The CNN holds abstracted data from patient charts by trained abstractors after discharge of patient from the neonatal intensive care unit (NICU) according to manual definitions. Data are cleaned at the coordinating center in Toronto and records with implausible data configurations are queried and corrected. The CNN holds individual patient-level data from all admissions to participating neonatal units in Canada from 1996. Since 2010, 25 of 28 NICUs in the country participated in data collection and, since 2012, all 30 units representing 100% coverage of those admitted to NICU in Canada. A formal reabstraction comparison has revealed it to be reliably reproducible. For the purposes of this study, infants with NE meeting National Institute for Child Health and Human Development (NICHD) cooling criteria were included. GBS disease was identified as GBS isolated from a sterile site (ie, blood, cerebrospinal fluid, or both).

## ESTIMATING THE UK INCIDENCE OF GROUP B *STREPTOCOCCUS*-ASSOCIATED NEONATAL ENCEPHALOPATHY

The number of term infants receiving ≥2 days cooling with GBS disease in England, Scotland, and Wales was identified from the NNRD database. Denominator data on term live births in England and Wales from 2012 to 2015 were identified from the UK Office for National Statistics [24]; and term live births in Scotland from 2014 to 2015 were identified from the National Records of Scotland [25]. These data were used to calculate the UK incidence of GBS-associated NE.

### Inclusion and Exclusion Criteria

For both published and unpublished data, we only considered original data including a denominator of at least 50 and we did not apply date or language restrictions (Supplementary Table 3). We included published and unpublished data for infants >35 weeks’ gestation with neonatal or HIE reporting on invasive GBS disease in the first 90 days after birth. Studies with nonrepresentative samples of cases and unsuitable article types such as case reports were excluded.

### Meta-analyses

Data on study characteristics and results were extracted into standardized prespecified Excel abstraction forms, and then imported to Stata 14 software (StataCorp) for meta-analyses. We used random-effects meta-analyses to estimate the proportion of infants with NE with GBS disease using the DerSimonian and Laird method [26]. Only datasets reporting the proportion of infants with GBS disease among those with NE assumed to be as a result of hypoxia-ischemia meeting the criteria for therapeutic hypothermia were included in the meta-analysis as this provided the most robust comparable denominator. Data from individual centers in the United Kingdom and Canada were not included in the meta-analysis due to overlap with the included national-level data. We conducted subgroup analysis for antenatal GBS screening practices.

### Death to Discharge and Longer-term Outcomes

Case fatality rates up until discharge among NE infants with and those without GBS disease were collected where available. Long-term outcome data, including mortality, cerebral palsy, and neurodevelopmental follow-up scores were sought. To estimate the difference between predischarge mortality for NE infants with and without GBS, we used a Mantel-Haenszel random-effects meta-analysis (RevMan 5.3) to generate the risk ratio (RR) and a *z* test to determine significance [27].

## RESULTS

### Literature and Investigator Group Data Inputs

**Figure 2.**
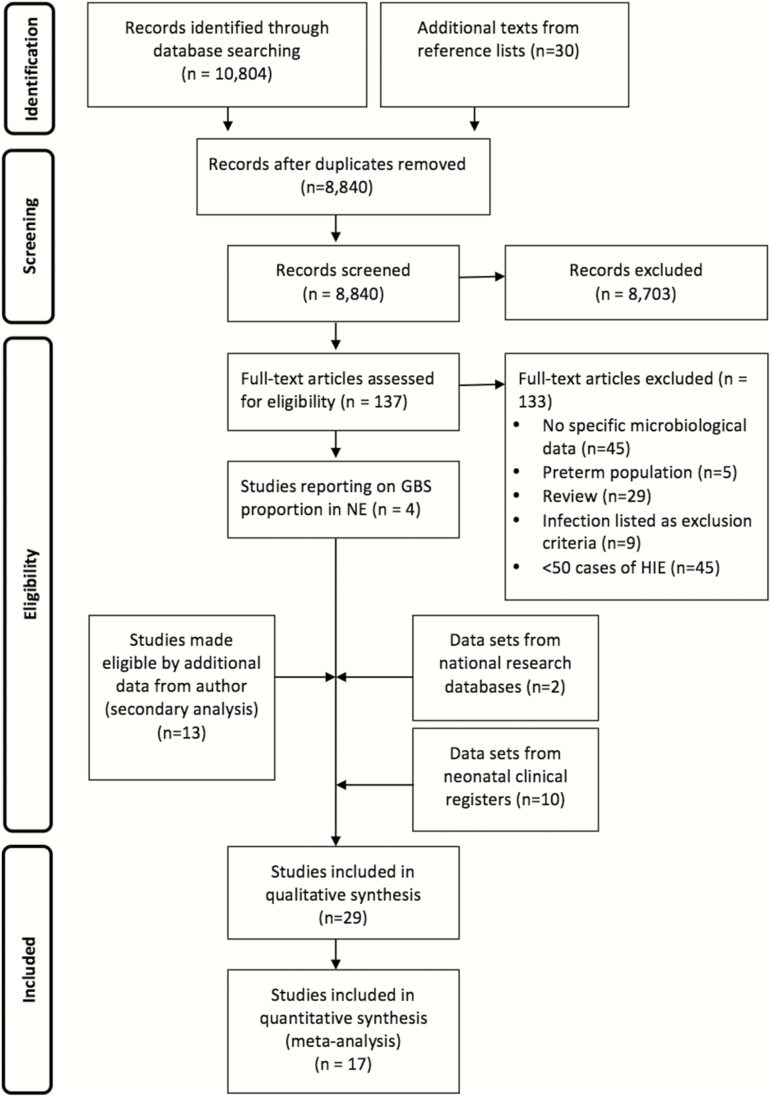
Search strategy and process of study selection regarding group B streptococcal disease among cases of neonatal encephalopathy. Abbreviations: GBS, group B *Streptococcus*; HIE, hypoxic-ischemic encephalopathy; NE, neonatal encephalopathy.

We identified 10804 studies through database searches. Of these, 137 full texts were reviewed and 4 studies met the inclusion criteria (Figure 2). Through the investigator group, an additional 13 were obtained from secondary analysis of published data, 10 from local cooling or neonatal neurology cohorts and 2 from national neonatal network research databases (Figure 2). Overall, 29 datasets met inclusion criteria; of these, 17 were eligible for inclusion in the meta-analysis. A total of 10436 infants were included; this number does not include datasets that either overlap with the United Kingdom or Canada national data, or the secondary analysis data from Jenster et al [20], which overlapped with the unpublished US cooling cohort dataset provided by H.C. Glass.

### Study Characteristics

**Figure 3.**
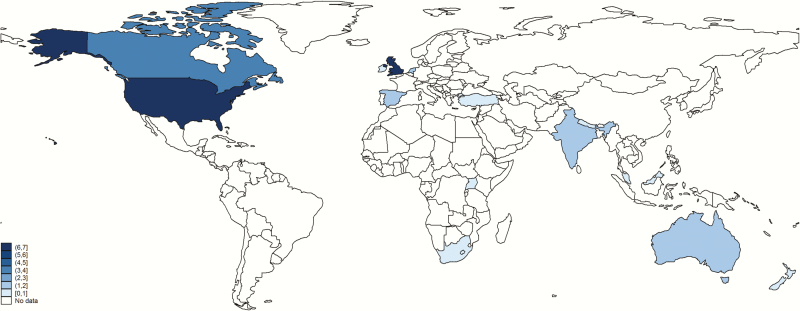
Geographic distribution of data inputs on neonatal encephalopathy with data on group B streptococcal disease. Borders of countries/territories in map do not imply any political statement.

Characteristics of published studies and unpublished datasets from our investigator group are shown in Table 1. Contributed data came from 13 countries (Figure 3). Only 4 published studies included data on GBS disease among infants with NE [19, 20, 28, 29]. Published and unpublished data were reported from both developed and developing countries (Figure 3); the majority are from HICs, in particular the United Kingdom, United States, and Canada. Of the 4 eligible published studies, 2 (1 US and 1 United Kingdom study) reported on blood culture results among a cohort of encephalopathic infants from the precooling era [20, 30]. A further published study from Turkey included culture data from infants meeting criteria for cooling among a cohort of encephalopathic infants [28]. The final published study was from a low-income setting, Uganda [19, 29]; the only cases (3) detected were detected with high cycle threshold values on species-specific real-time polymerase chain reaction [19]. National-level data were reported from 2 developed countries (United Kingdom and Canada). Secondary analyses of microbiological data were available from 4 cooling trials (CoolCap, TOBY Xenon, NICHD, and the Infant Cooling Evaluation Collaboration [ICE] trial [31–34]) and 9 encephalopathy cohorts [5, 6, 18, 29, 30, 35–38]. Unpublished datasets from our investigator group (10), reporting GBS disease among infants meeting criteria for cooling, were provided from 9 cooling registers from the United Kingdom, United States, Canada, Australia, and the Netherlands, with 2 further datasets for infants with NE who did not meet cooling criteria from Spain and Canada.

**Table 1.** Characteristics of Published and Unpublished Data Investigating Group B *Streptococcus*–Associated Neonatal Encephalopathy

Country	Year	Author			Type of Unit	Study Design	Published/Secondary Analysis/Unpublished	NE/HIE	Definition of NE/HIE	GA Range (Completed wk)	Birth Weight Range, g	BC Auto-mated	PCR	IAP	Death to Discharge	Included in Meta-analysis
Developed: NE/HIE meeting cooling criteria																
UK, national	2009–2016		NDAU		Tertiary referral	National neonatal database, retrospective	Unpublished	HIE	Term infants receiving ≥2 d of therapeutic hypothermia for treatment of NE	35–43	1280–6500	Y	N	Risk factor	Y	Y
Canada, national	2010–2015		CNN		Tertiary	National neonatal database, retrospective	Unpublished	HIE	NICHD cooling criteria^a^	35–44	1360–6770	Y	N	Screen	Y	Y
International, multisite (New Zealand, UK, US, Canada), CoolCap trial	1999–2003		Gunn, A		Tertiary referral	Cooling RCT prospective	Secondary analysis [28]	HIE	Cord or early neonatal pH <7.0, base excess > –16, & moderate or severe encephalopathy	Mean Cooled: 38.9 (SD, 1.6) Uncooled: 39.1 (SD, 1.4)	Mean Cooled: 3399 (SD, 663) Uncooled: 3504 (SD, 625)	Y	N	Majority Screen	Y	Y
US, multisite, NICHD cooling trial	2000–2003		Shankaran, S		Tertiary referral	Cooling RCT, prospective	Secondary analysis [31]	HIE	Meeting NICHD^a^ criteria for cooling	≥36 wk	Mean Cooled: 3385 (SD, 617). Not cooled: 3370 (SD, 645)	Y	N	Screen	Y	Y
Multisite (Australia, New Zealand, Canada, US), ICE trial	2001–2009		Jacobs, SE		Tertiary referral	Cooling RCT	Secondary analysis [30]	HIE	Grade 2/3 HIE (Sarnat), or at least 2 of the following: an Apgar ≤5 or at 10 min, continued need for mechanical ventilation at 10 min, cord or blood pH <7.00; or a base deficit of ≥12 within 60 min of birth	35–42	1978–5500	Y	N	Screen	Y	Y
UK, multisite, TOBY Xenon trial	2012–2014		Edwards, AD		Tertiary referral	Cooling RCT prospective	Secondary analysis [29]	HIE	TOBY cooling criteria^a^	36–43	Mean Cooled: 3213 (SD, 448) Cooled plus Xenon: 3392 (SD, 685)	Y	N	Risk factor	Y	N
US, Maryland	2007–2015		Johnson, CT		Tertiary referral	Cohort, retrospective	Secondary analysis [33]	HIE	Grade 2 or 3 HIE according to Sarnat cord gas or neonatal gas at <1 h with pH ≤7.0, a base deficit >16 mM, pH 7.01–7.15, and base deficit 10–15.9 mM (if moderate-severe encephalopathy was present with evidence of an acute sentinel event and a 10-min Apgar score <5), need for assisted ventilation that was initiated at birth for >10 min	34–41	1900–4920 g (average 3199 g)	Y	N	Screen	Y	Y
Spain, Barcelona	2009–2011		Garcia-Alix, A		Tertiary referral	Cohort, prospective	Secondary analysis [32]	HIE	Infants meeting the 3 following criteria: (1) altered fetal heart rate pattern, sentinel event, or labor dystocia; (2) Apgar score ≤5 at 10 min, or need for resuscitation, including endotracheal intubation or mask ventilation for >10 min after birth, or acidosis (pH ≤7.0 and/or base deficit ≥16 mmol/L within 60 min from birth; (3) NE defined as a syndrome of neurologic dysfunction manifested by a subnormal level of consciousness with or without seizures (moderate or severe HIE) or palmary hyperexcitability (tremor, overactive myotatic reflexes, hypersensitivity to stimulation or startle responses)	≥34–42	2000–4090	Y	Y	Risk factor	Y	Y
Ireland, Dublin	2010–2015		Hayes, B		Tertiary referral	Cohort, prospective	Secondary analysis [15]	HIE	TOBY cooling criteria^a^	34–41	2560–4010	Y	Y	Risk factor	Y	Y
UK, Bristol	2007–2016		Thoresen M		Tertiary referral	Cohort, prospective	Unpublished	HIE	TOBY cooling criteria^a^	>35	3360–3800	Y	N	Risk factor	Y	N
US, California	2008–2015		Glass, HC		Tertiary referral	Cohort, retrospective	Unpublished	HIE	All 3 criteria: (1) >36 wk, <6 h; (2) Apgar score <5 at 10 min, prolonged resuscitation at birth pH <7.00 and/or base excess < –12 (cord or blood gas) within 1 h; (3) moderate to severe encephalopathy	35–41	1790–5520	Y	N	Screen	Y	Y
The Netherlands, Utrecht	2008–2010		Groenendaal, F		Tertiary referral	Cooling cohort	Unpublished	HIE	Adapted TOBY cooling criteria^a^	34–42	1750–6230	Y	N	Risk factor	Y	Y
US, Boston	2008–2017		Walsh, BH		Tertiary referral	Cohort, prospective	Unpublished	HIE	AAP cooling criteria^a^	35 –42	2090–4150	Y	N	Screen	Y	Y
US, Washington, DC	2008–2016		Massaro, AN		Tertiary referral	Cohort, prospective	Unpublished	HIE	NICHD cooling criteria^a^	34–43	1787–6375	Y	N	Screen	Y	Y
Canada, Montreal	2008–2017		Wintermark, P		Tertiary referral	Cohort, retrospective	Unpublished	HIE	NICHD cooling criteria^a^	34–42	1930–6040	Y	N	Risk factor	Y	N
UK, London	2010–2016		Tann, CJ, Robertson, NJ		Tertiary referral	Cohort, prospective	Unpublished	HIE	TOBY cooling criteria ^a^	35–42	1765–5370	Y	N	Risk factor	Y	N
Australia, Melbourne	2010–2016		Cheong, J		Tertiary	Cohort, retrospective	Unpublished	HIE	≥35 wk gestation and at least 2 of the following; an Apgar score of ≤5 at 10 min, continued need for mechanical ventilation at 10 min, and/or cord or blood pH <7.00/ base deficit ≥12 within 60 min of birth	35–41	2050–5200	Y	N	Risk factor	Y	Y
Spain, Barcelona	2010–2016		Arca Diaz, G		Tertiary referral	Cooling cohort	Unpublished	HIE	TOBY cooling criteria^a^	34 + 1–43	2010–4200	Y	N	Risk factor	Y	Y
Developed: NE not meeting cooling criteria																
UK, London/ Netherlands, Utrecht	1992–1998			Cowan, F De Vries, LS	Tertiary referral	Cohort, prospective	Published [27]	NE	Abnormal tone pattern, feeding difficulties, altered alertness, and at least 3 of the following: (1) late decelerations on fetal monitoring or meconium staining; (2) delayed onset of respiration; (3) arterial cord blood pH <7.1; (4) Apgar scores <7 at 5 min; (5) multiorgan failure	37–42	2000–4900	Y	N	Risk factor	N	N
UK, London	1992–2007			Martinez- Biarge, M Cowan, F	Tertiary referral/ multiple local units	Cohort, prospective	Secondary analysis [5]	NE	Signs of intrapartum fetal distress and poor condition at birth (5-min Apgar score <5 and/or cord pH <7.1 and/or need for resuscitation) and early encephalopathy (mild, moderate, or severe)	35 + 1–43	1920–4600	Y	N	Unknown	Y	N
US, California	1993–2011			Jenster, M	Tertiary referral	Cohort	Published [17]	NE	One of the following: (1) first blood gas or umbilical cord artery pH <7.1; (2) first blood gas or umbilical cord artery base deficit >10; and/or (3) 5-min Apgar score ≤5	≥36	NA	Y	N	Screen	N	N
Canada, Montreal	2008–2016			Wintermark, P	Tertiary referral	Cohort, prospective	Unpublished	NE	NE, not meeting NICHD criteria	35–42	1920–5215	Y	N	Screen	Y	N
Asia																
Turkey, Ankara	2011–2013			Okomus, N	Tertiary referral	Cooling cohort	Published [25]	HIE	TOBY cooling criteria^a^	36–41	Mean 3175 (SD, 576)	Y	N	None	N	Y
Malaysia, multi-site	2012			Boo, N-Y	37 NICUs	Cohort, retrospective	Secondary analysis [31]	HIE	All 3 criteria: (1) any 3 features of encephalopathy within 72 h of birth; (2) 3 or more findings of acute perinatal events, eg, arterial cord pH <7.00, Apgar score <5 at 5 min of life, evidence of multiorgan system dysfunction <72 h of birth, evidence of fetal distress, abnormal electroencephalogram, and abnormal imaging of the brain showing ischemia or edema within 7 days of birth; (3) absence of any underlying congenital cerebral infections/abnormalities or inborn errors of metabolism that could account for the encephalopathy	>=36	Mean 3065 (SD, 486)	Y	N	Risk factor	Y	Y
India, Kerala, Peacock trial	2009			Thayyil, S	Tertiary	RCT, prospective	Secondary analysis [26]	NE	Infants requiring resuscitation at birth and/or Apgar score <5 at 5 min after birth and a Thompson encephalopathy score >5 within 6 h after birth	36–40	1950–3940	Y	N	None	Y	N
Nepal, Kathmandu	1995–1996			Ellis, M	Tertiary referral	Cohort, prospective	Secondary analysis [6]	NE	Fenichel modified criteria^a^	>37	1500–3999	N	N	N	N	N
India, multisite, Helix feasibility Trial	2013–2015			Thayyil, S	Tertiary	Cohort, prospective	Unpublished	NE	All 3 criteria: (1) age <6 h, birthweight >1.8 kg, gestation >36 wk; (2) need for resuscitation at 5 min and/or 5-min Apgar <6, or lack of cry by 5 min of age; (3) moderate/severe encephalopathy at <6 h of age on structured NICHD examination	36–42	2280–3800	Y	N	None	Y	N
Africa																
South Africa, Cape Town	2008–2011			Kali, G	Tertiary	Cohort, retrospective	Secondary analysis [34]	HIE	TOBY cooling criteria^a^	35–43	1960–5190	Y	N	Risk factor	Y	Y
Uganda, Kampala	2010–2011			Tann, C	Tertiary referral	Case-control, prospective	Published [16]	NE	Term infants with Thompson score >5 within 12 h of birth^a^	>36	1940–4640	Y	Y	None	Y	N

Abbreviations: AAP, American Academy of Pediatrics; BC, blood culture; GA, gestational age; HIE, hypoxic-ischemic encephalopathy; IAP, intrapartum antibiotic prophylaxis; ICE, Infant Cooling Evaluation Collaboration; NA, not available; NE, neonatal encephalopathy; NICU, neonatal intensive care unit; NICHD, National Institute for Child Health and Human Development; PCR, polymerase chain reaction; RCT, randomized controlled trial; SD, standard deviation; TOBY, Total Body Hypothermia trial; UK, United Kingdom; US, United States.

^a^See Supplementary Table 4 for full criteria.

Therapeutic whole-body cooling became standard of care across many high-income-country settings from 2008 to 2009, meaning the majority of datasets included here are from 2008 onward. Commonly used criteria for therapeutic hypothermia are summarized in Supplementary Table 4. However, cooling trial data from as early as 1999 are also included from the CoolCap Trial [31]. Data are reported from settings with a variety of approaches to intrapartum antibiotic prophylaxis (IAP) for maternal GBS colonization including areas with active screening (eg, United States, Canada, Australia) to those using a risk factor–based approach (eg, the Netherlands, United Kingdom, and Ireland) and those with no defined national approach to IAP for early-onset GBS prevention (eg, Uganda, India, and Nepal). All data reported on infants born at >35 weeks completed gestational age and includes a wide range of birth weights (Table 1).

### Proportion of Group B *Streptococcus* Disease Among Infants With Neonatal Encephalopathy

Twenty-one studies reported on the proportion of GBS disease among infants with NE, assumed to be due to hypoxia-ischemia, meeting criteria for cooling (Table 2). An additional 8 studies reported on the proportion of GBS disease among infants with NE not meeting cooling criteria (Table 3). The proportion with GBS disease among all cohorts varied from 0 to 2.4%. The highest rates of GBS-associated NE were reported in cohorts from the United Kingdom, United States [34], Ireland [18], Canada, and Malaysia where the proportion with GBS disease in all was >1%. Cohorts from the United States [20], Australia, Spain [36], Turkey [28], India [29], and Nepal [6] reported no GBS disease among infants with NE. However, of the 10 datasets with 0 GBS cases, the number of births were 53, 54, 72, 74, 89, 90, 95128, 252, and 258, so the only 2 datasets powered to detect this low expected proportion were those from the same US center with a known high coverage of IAP [20].

**Table 2.** Proportion of Group B *Streptococcus* Among Cases With Neonatal Encephalopathy Assumed to Be Due to Hypoxia-Ischemia Meeting Criteria for Therapeutic Hypothermia

Country	Location	Year(s)	Author	Database	Denominator^a^	NE Cases	GBS Invasive Disease	% of NE With GBS
Developed								
UK	National	2009–2016	NDAU	National research database	HIE, cooling criteria	6041	72	1.19
Canada	National	2010–2015	CNN	National research database	HIE, cooling criteria	1184	2	0.17
International, multisite	CoolCap trial	1999–2003	Gunn, A	Cooling trial	HIE, cooling criteria	234	1	0.43
US, multisite	NICHD cooling trial	2000–2003	Shankaran, S	Cooling trial	HIE, cooling criteria	208	5	2.40
International, multisite	ICE Trial	2001–2007	Jacobs, SE	Cooling trial	HIE, cooling criteria	221	3	1.36
UK, multisite	TOBY Xenon trial	2012–2014	Edwards, AD	Cooling trial	HIE, cooling criteria	92^a^	2	2.17
US	Maryland	2007–2015	Johnson, CT	Cooling cohort	HIE, cooling criteria	57	1	1.75
Spain	Barcelona	2009–2011	Garcia-Alix, A	Cooling cohort	HIE, cooling criteria	53	0	0
Ireland	Dublin	2010–2015	Hayes, B	Cooling cohort	HIE, cooling criteria	76	1	1.31
UK	Bristol	2007–2016	Thoresen, M	Cooling register	HIE, cooling criteria	292^a^	2	0.68
US	San Francisco	2008–2015	Glass, HC	Cooling register	HIE, cooling criteria	252	0	0
The Netherlands	Utrecht	2008–2010	Groenendaal, F	Cooling register	HIE, cooling criteria	192	4	2.08
US	Boston	2008–2017	Walsh, BH	Cooling register	HIE, cooling criteria	72	0	0
US	Washington, DC	2008–2016	Massaro, AN	Cooling register	HIE, cooling criteria	187	2	1.07
Canada	Montreal	2009–2016	Wintermark, P	Cooling register	HIE, cooling criteria	253^a^	3	1.18
UK	London	2010–2016	Tann, CJ Robertson, NJ	Cooling register	HIE, cooling criteria	256^a^	6	2.34
Australia	Melbourne	2010–2016	Cheong, J	Cooling register	HIE, cooling criteria	128	0	0
Spain	Barcelona	2010–2016	Arca-Diaz, G	Cooling register	HIE, cooling criteria	90	0	0
Asia								
Turkey	Ankara	2011–2013	Okomus, N	Cooling cohort	HIE, cooling criteria	74	0	0
Malaysia	Multisite	2012	Boo, NY	Cooling cohort	HIE, cooling criteria	919	10	1.09
Africa								
South Africa	Cape Town	2008–2011	Kali, G	Cooling cohort	HIE, cooling criteria	94	1	1.06

Abbreviations: CNN, Canadian Neonatal Network; GBS, group B *Streptococcus*; HIE, hypoxic-ischemic encephalopathy; ICE, Infant Cooling Evaluation Collaboration; NDAU, Neonatal Data Analysis Unit; NE, neonatal encephalopathy; NICHD, National Institute of Child Health and Human Development; TOBY, Total Body Hypothermia; UK, United Kingdom; US, United States.

^a^Cases not included in the total denominator, where there is overlap with national data or another dataset.

**Table 3.** Proportion of Group B Streptococcal Disease Among Cases of Neonatal Encephalopathy

Country	Location	Year	Author	Database	Denominator	Cases	GBS Invasive Disease	% of NE With GBS
Developed								
UK/ Netherlands	London/ Utrecht	1992–1998	Cowan, F de Vries, LS	Published	NE, not meeting cooling criteria	253^b^	2	0.79
UK	London	1992–2007	Martinez-Biarge, MCowan, F	Published	NE, not meeting cooling criteria	259^b^	5	1.93
US	San Francisco	1993–2011	Jenster, M^a^	Published	NE, clinical criteria	258^b^	0	0
Canada	Montreal	2009–2016	Wintermark, P	Neonatal neurology register	NE, not meeting cooling criteria	249^b^	1	0.40
Asia								
India	Kerala	2009	Thayyil, S	Cooling cohort	NE, clinical criteria	54	0	0
Nepal	Kathmandu	1995–1996	Ellis, M	Observational study data	NE, clinical criteria	95	0	0
India	Multisite	2013–2015	Thayyil, S	Observational study data	NE, clinical criteria	89	0	0
Africa								
Uganda	Kampala	2011–2012	Tann, CJ	Case-control study	NE, clinical criteria	210	3	1.43

Abbreviations: GBS, group B Streptococcus; NE, neonatal encephalopathy; UK, United Kingdom; US, United States.

^a^The only baby reported to be GBS positive was found to be group A *Streptococcus* positive; erratum awaiting publication.

^b^Number of cases not included in the total denominator, where there is overlap with national data or another dataset.

### Meta-analysis of the Proportion of Group B *Streptococcus* Disease Among Infants Meeting Criteria for Therapeutic Hypothermia

**Figure 4.**
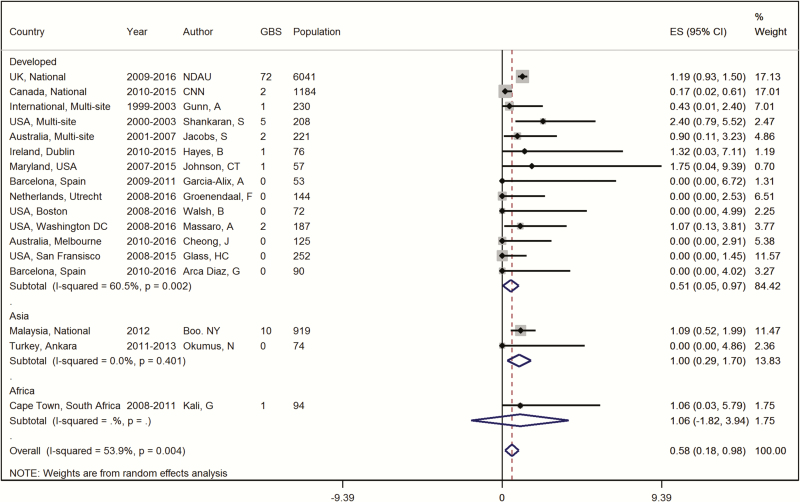
Meta-analysis of the proportion of group B *Streptococcus* identified from a sterile site among infants with neonatal encephalopathy assumed to be due to hypoxic-ischemic encephalopathy, in infants meeting criteria for therapeutic hypothermia. Abbreviations: CI, confidence interval; CNN, Canadian Neonatal Network; ES, estimate; GBS, group B *Streptococcus*; NDAU, Neonatal Data Analysis Unit.

Seventeen datasets were eligible for inclusion in the meta-analysis examining the proportion of GBS disease among infants with encephalopathy meeting criteria for therapeutic hypothermia (Figure 4). Three UK datasets and 4 Canadian datasets were excluded from the meta-analysis because the data overlapped with the included national neonatal data from the same countries. Data inputs were all from HICs in the UN “developed” region, with the exception of Malaysia and South Africa, both upper-middle–income countries. No studies from low-income countries (LICs) were eligible for inclusion in the meta-analysis as diagnostic techniques required for cooling criteria such as cord and blood gas estimation and cerebral function monitoring are largely unavailable. Among those included in the meta-analysis, the proportion with GBS disease among infants with NE meeting cooling criteria was 0.58% (95% confidence interval [CI], .18%–.98%; range, 0–2.40%) (Figure 4). Subgroup analysis by antenatal screening practice demonstrated a proportion with GBS disease of 1.09 (95% CI, .84–1.35) for datasets without an antenatal GBS screening policy, compared with 0.21 (95% CI, .00–.48) for datasets where antenatal screening was routine (Supplementary Figure 1). One dataset (CoolCap) was excluded from this subgroup analysis due to varying screening practices across multiple sites.

### Death to Discharge and Longer-Term Outcomes

Studies reporting on case fatality among infants with NE, with and without GBS disease, are presented in Table 4. Case fatality to discharge among infants with NE was reported in 24 studies: 2 from national research data (United Kingdom, Canada), 3 from secondary analysis of cooling trial data (CoolCap [31], NICHD [34], and ICE [33]), and 19 from NE cohorts. However, of these, 8 studies reported no GBS disease in their NE cohort, and 4 overlapped with national data. The remaining 12 datasets were used to compare the case fatality in GBS-associated NE and NE alone. Case fatality before discharge for these 12 datasets combined was 1305 of 9525 (13.7%) infants with NE alone compared with 22 of 105 (21%) infants with NE and GBS (*P* < .0001). Risk of mortality before discharge was significantly increased in those infants with GBS-associated NE compared to those without GBS disease (risk ratio [RR], 2.07 [95% CI, 1.47–2.91]; Supplementary Figure 2). A sensitivity analysis, excluding cohorts with the lowest GBS incidence, did not markedly alter this RR. Longer-term outcome data were consistently reported as either incomplete or not available and so are not reported here.

**Table 4.** Case Fatality at Discharge Among Infants With Neonatal Encephalopathy With and Without Group B Streptococcal Disease

Country	Location	Author	Denominator	Case Fatality Among Infants With NE, no./No. (%)		
				Overall	With Invasive GBS	No Invasive GBS
Developed						
UK	NNRD	NDAU	HIE, cooling criteria	638/6041 (10.6)	9/72 (12.5)	629/5969 (10.5)
Canada	National	CNN	HIE, cooling criteria	211/2250 (9.3)	3/4 (75.0)	208/2246 (9.3)
International	Multisite	Gunn, A	HIE, cooling criteria	58/234 (24.8)	1/1 (100.0)	57/233 (24.5)
US	Multisite	Shankaran, S	HIE, cooling criteria	62/208 (29.8)	2/5 (40)	60/203 (29.6)
International	Multisite	Jacobs, SE	HIE, cooling criteria	58/221 (26.2)	1/3 (33.3)	57/218 ((26.1)
US	Maryland	Johnson, CT	HIE, cooling criteria	3/57 (5.26)	0/1 (0)	3/56 (5.36)
Spain	Barcelona	Garcia-Alix, A	HIE, cooling criteria	16/51 (31.4)	0/0 (0)	16/51 (31.4)
Ireland	Dublin	Hayes, B	HIE, cooling criteria	17/76 (22.4)	1/1 (100)	16/75 (21.3)
US	California	Glass, HC	HIE, cooling criteria	26/252 (10.32)	0/0 (0)	26/252 (10.32)
The Netherlands	Utrecht	Groenendaal, F	HIE, cooling criteria	63/192 (32.8)	0/4 (0)	63/192 (32.8)
US	Boston	Walsh, BH	HIE, cooling criteria	3/72 (4.2)	0/0 (0)	3/72 (4.2)
US	Washington, DC	Massaro, AN	HIE, cooling criteria	30/187 (16.0)	1/2 (50.0)	29/185 (15.7)
Canada	Montreal	Wintermark, P	HIE, cooling criteria	36/253 (14.2)	0/4 (0)	36/249 (14.5)
UK	London	Tann, CJRobertson, N	HIE, cooling criteria	30/187 (16.0)	1/2 (50.0)	29/185 (14.7)
Australia	Melbourne	Cheong, J	HIE, cooling criteria	34/128 (26.5)	0/0 (0)	34/128 (26.5)
Spain	Barcelona	Arca-Diaz, G	HIE, cooling criteria	8/90 (8.89)	0/0 (0)	8/90 (8.89)
UK	London	Martinez-Biarge, MCowan, F	NE, clinical criteria	22/259 (8.50)	0/5 (0)	22/259 (8.50)
Canada	Montreal	Wintermark, P	NE, clinical criteria	6/249 (2.4)	0/1 (0)	6/248 (2.4)
Asia						
Malaysia	Multisite	Boo, N-Y	HIE, cooling criteria	144/919 (15.6)	4/10 (40.0)	140/909 (15.4)
Nepal	Kathmandu	Ellis, M	NE, clinical criteria	40/142 (28.2)	0/0 (0)	40/142 (28.2)
India	Kerala,	Thayyil, S	NE, clinical criteria	6/54 (11.1)	0/0 (0)	6/54 (11.1)
India	Multisite	Thayyil, S	NE, clinical criteria	16/89 (18.0)	0/0 (0)	16/89 (18.0)
Africa						
South Africa	Cape Town	Kali, G	HIE, cooling criteria	14/99 (14.1)	0/1 (0)	14/98 (14.3)
Uganda	Kampala	Tann, CJ	NE, clinical criteria	70/208 (33.7)	2/3 (66.6)	68/205 (33.2)

Abbreviations: CNN, Canadian Neonatal Network; GBS, group B Streptococcus; HIE, hypoxic-ischemic encephalopathy; NDAU, Neonatal Data Analysis Unit; NE, neonatal encephalopathy; NNRD, UK National Neonatal Research Database; UK, United Kingdom; US, United States.

### UK Population-Level Data

The number of infants with GBS-associated NE in England and Wales between 2012 and 2015 was 55 and the number of term live births identified over the same period in England and Wales, according to the Office for National Statistics, was 2821271 [24]. The number of infants with GBS-associated NE in Scotland between 2014 and 2015 was 1 and the number of term live births identified over the same period in Scotland was 111823 [25]. Northern Ireland population-level NE data were not available. The UK incidence of GBS-associated NE was therefore 0.019 (95% CI, .019–.02) per 1000 live births.

## DISCUSSION

This systematic review and meta-analysis is the first to estimate the percentage of GBS disease in neonates with NE and to assess differences in survival outcomes compared with infants with NE alone. Overall published data are lacking; however, by including national research databases, secondary analyses, and data from neonatal neurology registers we have been able to include comparable data from 29 centers, across 13 countries. The contribution from a large number of investigator groups across the globe has provided an extensive dataset for this paper addressing GBS disease with NE. Due to the sizeable dataset denominator, we are able to more accurately determine the proportion of NE associated with GBS, and estimate differences in mortality outcome. Our findings show that infants with NE are >10 times more likely to be affected by invasive GBS disease than term infants without NE. The national UK data also enabled the first population-wide incidence estimate of GBS-associated NE, which has contributed to our understanding of the global burden of GBS disease [39].

The contribution of the UK NNRD and Canadian CNN national data have enabled inclusion of every infant admitted to the NICU with NE fulfilling criteria for therapeutic hypothermia in these 2 countries. In addition, the systematic review, performed to identify published data on GBS-associated NE, is comprehensive, reproducible, and was conducted on multiple databases, without limitation by language. A range of search terms to describe NE were utilized to allow for shifting terminology and definitions. Our findings show that 0.58% (95% CI, .18%–.98%) of infants with NE who meet criteria for therapeutic hypothermia have GBS disease. This is >10-fold higher than the 0.046% (95% CI, .038%–.053%) developed region estimate of GBS disease for all term liveborn infants [40].

The incidence of GBS-associated NE ranged from 0% to 2.4%, without restriction to those achieving cooling criteria. There was notable disparity between the 2 largest datasets—the UK and Canada national research databases had 1.19% and 0.17% GBS disease, respectively. The variation is possibly a reflection of the low overall incidence of identified GBS-associated NE; however, it is important to consider the role of intrapartum GBS prophylaxis. Our meta-analysis included data from countries that routinely screen and treat for maternal GBS colonization (United States, Canada, Australia), countries that take a risk factor approach to screening (UK, Ireland, Malaysia, Spain, South Africa) and countries with no current policy (Turkey). GBS disease was more frequently found in NE infants receiving cooling in countries that do not routinely screen mothers (proportion with GBS, 1.09% [95% CI, .84%–1.35%]) compared to other countries that do (proportion with GBS, 0.21% [95% CI, .00%–.48%]). It is notable that a number of NE cohorts from India and Nepal, with no reported policy for GBS prophylaxis, had no infants with GBS-associated NE. It is unclear why these cohorts have zero case ascertainment, although early death in settings where neonatal intensive care is not available may have contributed. The US NICHD dataset has the largest incidence of GBS (2.4%) [34] and is an outlier among others in the subgroup with antenatal GBS screening.

Meta-analysis of the proportion of GBS identified from a sterile site among infants with NE is only a measure of the proportion of NE with GBS disease. From a public health perspective, to obtain a population incidence of GBS-associated NE also necessitates data regarding the incidence of NE per 1000 live births. UK national data [24, 25] can provide population-wide estimates of GBS associated NE. We estimate that GBS-associated NE occurs in 0.019 per 1000 overall live births (95% CI, .019–.02 per 1000 live births).

The risk of death before discharge was doubled for infants with a combination of NE and GBS disease, compared with NE alone (GBS-associated NE mortality was 21% compared with 13.7% for NE alone; RR, 2.07 [95% CI, 1.47–2.91]; *P* < .0001). This must be interpreted cautiously in view of the small denominator size among the GBS-associated NE group. The increased deaths may have been due to overwhelming sepsis; however, it is also biologically plausible that sensitization by inflammation increased the extent of brain injury relative to the severity of an ischemic insult alone, as demonstrated in preclinical models [14, 41]. Systemic illness for infants affected with infection and hypoxia-ischemia is likely to be more severe, with a combination of hypoxic injury and inflammatory cascade–induced dysfunction of organ systems. In one clinical HIE cohort of 258 infants, evidence of neonatal sepsis was associated with a significant increase in brain injury on neuroimaging and a trend toward increased mortality and neurodevelopmental impairment [20]. Importantly, in inflammation-sensitized HIE preclinical models, hypothermia has been shown to be variably neuroprotective [17, 42]. A recent study, modeling gram-positive infection prior to HI injury, demonstrated sensitization of the brain to HI injury, but also encouragingly, neuroprotection with hypothermia [40]. Therapeutic hypothermia may also contribute to systemic instability in sepsis (eg, independently lowers blood pressure) [43]. Additionally, therapeutic hypothermia causes chemokine-associated immunosuppression, which reduces peripheral leukocyte numbers [44] and may impair immune responsiveness to GBS. Reassuringly, treatment with therapeutic hypothermia was not associated with an increase in culture-positive sepsis in a large meta-analysis of therapeutic hypothermia efficacy [9]. The possible higher risk of mortality with GBS-associated NE, and the difficulties in differentiating sepsis, highlights the importance of empiric antibiotics for all infants with NE while investigations for concomitant infection are ongoing or where infection cannot easily be ruled out.

### Paucity of Data Especially in Low- and Middle-Income Countries

There were no published data specific to GBS-associated NE, and only 4 published studies reporting on GBS incidence in NE cohorts. This lack of data was overcome, in part, through the participation of the many contributions through our investigator group.

NE is estimated to be the cause in 23% of neonatal deaths worldwide, with 99% of all neonatal deaths occurring in LMICs [45]. The incidence of intrapartum-related NE has been estimated to be 14.9 per 1000 live births in LMICs compared to 1.6 per 1000 live births in HICs [2], with 95% of global death and impairment secondary to intrapartum-related events occurring in LMICs [2]. The meta-analysis of GBS-associated NE among infants does not include any data from LICs, where therapeutic hypothermia is not in widespread use, and any published data were from prohibitively small cohorts. It can be postulated, however, that the burden of GBS disease in combination with NE is likely to be greater in LIC settings and our estimates of the incidence of GBS-associated NE are likely to be an underrepresentation of the global problem.

### Paucity of Data for Long-Term Impairment

Death before discharge was the only available outcome measure examined. Ideally, short-term outcomes that have been found to be predictive of longer-term outcomes, such as magnetic resonance imaging (MRI), would also be reported. More importantly, there were insufficient follow-up data available to determine long-term outcome, and these data are generally lacking after NE [2], after neonatal infections [22], and notably after GBS [46]—from which this group of infants with GBS-associated NE can be considered distinct. Investigator groups reported a lack of consistent follow-up of these infants as the reason for the paucity of data. If, as preclinical studies suggest, there is increased neuronal injury in newborns with inflammation-sensitized HIE, the risk of neurodevelopmental impairment and later mortality is likely to be increased. In 1 case-control study, cerebral palsy was strongly associated with a combination of clinical chorioamnionitis and MRI evidence of hypoxic-ischemic injury (odds ratio, 17.5 [95% CI, 3.3–93.4]; *P* = .001) [47].

### Paucity of Data in Stillbirths

Stillborn infants are an important group of infants not represented in our analysis. Recent analyses show the important contribution of infections to the global toll of 2.6 million annual stillbirths [48], but data on GBS and stillbirth are limited [49]. Combined hypoxia-ischemia and GBS disease during labor may result in even more intrapartum stillbirths than neonatal deaths as preclinical models demonstrate an inability by inflammation-sensitized newborns to survive a hypoxic-ischemic insult [41, 42].

### Neonatal Encephalopathy Case Definition and Subsequent Bias

NE is a descriptive term for a constellation of clinical features, without ascribing cause [50]. Terms such as birth asphyxia, perinatal asphyxia, HIE, and NE are often interchangeably and incorrectly used [2]. To address this in the meta-analysis, and ensure the denominator was comparable between cohorts, evidence of fulfilling the relatively comparable criteria for therapeutic hypothermia was used. However, the criteria for cooling were designed to specifically identify that subgroup of infants with NE where hypoxia-ischemia is assumed to be the primary etiology. However, as a result of this, infants with NE due to causes other than intrapartum asphyxia will be underrepresented in our analysis. Septic infants presenting with NE (abnormal neurological symptoms are present in 63% of neonates with GBS disease [51]) will not be included in the meta-analysis unless they also had evidence of intrapartum asphyxia. Additionally, infants with overwhelming sepsis may be excluded from cooling even in the presence of HIE.

For the UK NNRD dataset, only infants cooled for ≥2 days were included in analysis. This was to ensure exclusion of infants without clinical evidence of moderate to severe HIE, who were subsequently rewarmed early. As a result, cases will be missed, namely, those that died on the first day or were rewarmed early due to severity of systemic illness.

Cohorts with NE cases not specifying fulfilment of cooling criteria were excluded from meta-analysis; however, this excluded data from LICs. This selection bias to our estimate is likely to underrepresent the true incidence of GBS-associated NE since the incidence of NE has been reported to be 10 times higher in low-resource settings [2], and infectious comorbidity is also likely to be higher.

### Group B *Streptococcus* Case Ascertainment

Diagnosing GBS invasive disease is an important challenge, as outlined in elsewhere in this supplement [52]. The yield of cultures is recognized to be low in neonates due to prior receipt of intrapartum antibiotics; small specimen volume; and prioritization of rapid administration of postnatal antibiotics over sampling, especially for lumbar punctures. “Culture-negative” sepsis is a well-recognized entity in neonatal medicine [53]. Polymerase chain reaction testing for GBS is available but not widely adopted yet. GBS isolated from the skin, mucosa, trachea, or urine was not included in this review due to the uncertainty over colonization vs infection. The number of cases of GBS invasive disease is therefore likely underrepresented.

### Improving the Data

The paucity of data on concomitant infection in NE, and long-term outcomes for infants with NE needs to be addressed. It is notable that most major cooling collaboratives reported that they do not routinely collect data or report on GBS or other coinfection; given the likely contributory role and increase in mortality, we recommend this be implemented for GBS and for other peripartum pathogens such as gram-negative organisms. Future trials of neuroprotective adjuncts to therapeutic hypothermia should incorporate data collection relevant to intrapartum and neonatal infection, and consider secondary analysis split by presence of infection. Importantly, long-term neurodevelopmental follow-up of all NE infants should be standard practice, both for clinical care and for quality improvement purposes. Addressing data gaps in LMICs should be prioritized.

### Public Health Implications

Our conservative estimate of GBS disease occurring in only 0.58% of NE cases has a larger impact when applied on a global scale. Worldwide there are an estimated 1.16 million cases of NE per year (8.5 per 1000 live births), resulting in a quarter of neonatal deaths [45] and in neurodevelopmental impairment in 700000 children per year [2]. Preventing GBS infection will likely reduce global NE incidence and mortality. IAP may go some way to reduce infection but will not always be implemented prior to in utero infection/inflammation onset. More important, IAP is not available to all women around the world. For these reasons, maternal vaccination against GBS is endorsed.

## CONCLUSIONS

This meta-analysis highlights the importance of recognizing GBS infection in infants with NE. The proportion of term infants with NE and coexisting GBS infection is >10 times that seen among term infants without NE. Mortality rate among infants with GBS-associated NE is close to double that of infants with NE without GBS. The final estimate of GBS invasive disease in association with NE is limited to a subset of encephalopathic infants in high-resource settings, fulfilling criteria for therapeutic hypothermia, and is therefore likely an underestimation of the global situation, especially in low-resource settings where access to intrapartum care is lacking. Robust follow-up data were not available to determine the impact of GBS infection in combination with NE on long-term survival and neurodevelopmental impairment. To ascertain the full extent of the burden of disease, data gaps must be addressed, including long term follow-up of NE survivors, especially those with infection, and increased data from LMICs. The increased mortality rate in GBS-associated NE is unlikely to be completely addressed by IAP and may be more effectively prevented by maternal vaccination (Table 5).

**Table 5.** Key Findings and Implications

What’s new about this? • NE is a major cause of child mortality and long-term impairment. There is increasing evidence of a sensitizing role of in utero infection. GBS is an important perinatal pathogen, yet there was limited published data regarding its role in NE. • This is the first systematic review and meta-analysis of GBS-associated NE. Previously unpublished data were sourced from 25 cohorts, across 13 countries via our investigator groups, to include 10436 infants with NE.
What was the main finding? • 0.58% (95% CI, .18%–.98%) of NE cases are associated with GBS which is >10 times that in term infants without NE. • GBS-associated NE is associated with an increased risk of mortality compared with NE alone (2.07 [95% CI, 1.47–2.91]). • The incidence of GBS-associated NE in the United Kingdom is 0.019% (95% CI, .019%–.02%) per 1000 live births.
How can the data be improved? • Systematic collection of data regarding evidence of intrapartum or neonatal infection (GBS and other organisms) for all therapeutic hypothermia registries, future neuroprotection trials, and neonatal national databases. • Address the data gaps for LMICs. • Consider GBS PCR testing of blood and CSF to increase diagnostic yield.
What does it mean for policy and programs? • Addressing intrapartum infection is important for program prevention strategies for NE and subsequent neonatal death and neurodisability; as well as for intrapartum stillbirth. • The contribution of GBS to these outcomes is relevant in considering maternal GBS vaccination. • A GBS vaccination program may be advantageous compared to IAP due to accessibility in low-resource settings, plus earlier prevention of unrecognized in utero infection.

Abbreviations: CI, confidence interval; CSF, cerebrospinal fluid; GBS, group B Streptococcus; IAP, intrapartum antibiotic prophylaxis; LMIC, low- to middle-income country; NE, neonatal encephalopathy; NNRD, UK National Neonatal Research Database; PCR, polymerase chain reaction.

## Supplementary Data

Supplementary materials are available at *Clinical Infectious Diseases* online. Consisting of data provided by the authors to benefit the reader, the posted materials are not copyedited and are the sole responsibility of the authors, so questions or comments should be addressed to the corresponding author.

## Supplementary Material

Supplement-material

## Notes

***Author contributions.*** The concept of the estimates and the technical oversight of the series was led by J. E. L. and A. C. S. The reviews, analyses, and first draft of the paper were undertaken by C. J. T. with P. T. H., K. M., and S. Sad. Other specific contributions were made by J. E. L., A. C. S., M. V. P., and N. R.. The GBS Estimates Expert Advisory Group (C. J. B., L. B., C. C., M. G. G., M. I., K. L. D., S. A. M., C. E. R., S. K. S., S. Sc., A. S.-t. M., J. V.) contributed to the conceptual process throughout, notably on the disease schema and data inputs. The GBS Neonatal Encephalopathy Investigator Group (see above) input data for the analyses. All the authors reviewed and contributed to the manuscript. C. T. and P. H. had full access to all the data in the study and take full responsibility for the integrity of the data and accuracy of the data analysis.

***Acknowledgments.*** We are grateful for the time and efforts of the investigator group, their research teams, and supporting personnel who recorded data. Thanks also go to the families that agreed to the inclusion of their baby’s data in the investigator group datasets including the NNRD. At London School of Hygiene & Tropical Medicine (LSHTM), thanks go to Fiorella Bianchi-Jassir and Claudia da Silva for administrative assistance and Alegria Perez for coordinating author signatures. Finally, our thanks go to the Expert Advisory Group for their advice and support.

***GBS Neonatal Encephalopathy Investigator Group.*** Alfredo Garcia-Alix (Hospital Sant Joan de Déu, University of Barcelona, Spain), Nem-Yun Boo (Universiti Tunku Abdul Rahman, Malaysia), Miriam Martinez-Biarge (Imperial College London, UK), Jeanie Cheong (Royal Women’s Hospital Melbourne, Murdoch Children’s Research Institute, University of Melbourne, Australia), Frances Cowan (Imperial College, Hammersmith Hospital, London, UK), Linda S. de Vries (University Medical Center Utrecht, The Netherlands), Gemma Arca-Diaz (Hospital Sant Joan de Déu, University of Barcelona, Spain), A. David Edwards (Kings College London, UK, MRC Centre for Neurodevelopment Disorders, UK, St Thomas’ Hospital Trust, London, UK), Matthew Ellis (Imperial College London, UK), Christopher Gale (Imperial College, London, UK), Hannah C. Glass (University of California, San Francisco), Floris Groenendaal (University Medical Center Utrecht, The Netherlands), Alistair Gunn (University of Auckland, Auckland, New Zealand), Breda Hayes (Rotunda Hospital, Ireland), Susan E. Jacobs (Royal Women’s Hospital Melbourne, Murdoch Childrens’ Research Institute, Melbourne, Australia), Clark T. Johnson (Johns Hopkins University School of Medicine, Baltimore, Maryland), Gugu Kali (Tygerberg Hospital/ Stellenbosch University, South Africa), Manogna Manne (University of California, Los Angeles), An N. Massaro (Children’s National Medical Center/George Washington University School of Medicine, Washington, DC), Nicola J. Robertson (University College London, UK), Prakeshkumar Shah (Mount Sinai Hospital and University of Toronto, Canada), Seetha Shankaran (Wayne State University/Children’s Hospital of Michigan, Detroit), Cally J. Tann (LSHTM, UK, University College London Hospital, UK), Sudhin Thayyil (Imperial College, UK, Imperial Neonatal Service, UK), Marianne Thoresen (University of Bristol, UK, University of Osla, Norway, St Michaels Hospital, Bristol, UK), Brian H. Walsh (Brigham and Women’s Hospital, Boston, Massachusetts), Pia Wintermark (McGill University, Montreal, Canada), Anne C. C. Lee (Department of Pediatric Newborn Medicine, Brigham and Women’s Hospital, Harvard Medical School, Boston, Massachusetts).

***Financial support.*** This supplement was supported by a grant to the London School of Hygiene & Tropical Medicine from the Bill & Melinda Gates Foundation (Grant ID: OPP1131158).

***Supplement sponsorship.*** This article appears as part of the supplement “The Burden of Group B *Streptococcus* Worldwide for Pregnant Women, Stillbirths, and Children,” sponsored by the Bill & Melinda Gates Foundation and coordinated by the London School of Hygiene & Tropical Medicine.

***Potential conflicts of interest.*** Many contributors to this supplement have received funding for their research from foundations, especially the Bill & Melinda Gates Foundation, and several from the Wellcome Trust, the Medical Research Council UK, the Thrasher Foundation, the Meningitis Research Foundation, and one individual from the US National Institutes of Health. Members of the Expert Advisory Group received reimbursement for travel expenses to attend working meetings related to this series. A. S.-t. M. works for the Bill & Melinda Gates Foundation. C. J. B. has served as a member of the Presidential Advisory Committee for Seqirus Inc and of the CureVac Inc Scientific Advisory Committee, as well as undertaken consultancy work for Pfizer Inc. C. C. has received institutional compensation from Novartis for conducting GBS studies. P. T. H. has been a consultant to Novartis and Pfizer on GBS vaccines but received no funding for these activities. M. I. has undertaken sponsored research from Pfizer on pneumococcal disease in adults and from Belpharma Eumedica (Belgium) on temocillin antimicrobial susceptibility in Enterobacteriaceae. K. L. D. has received funding by the Bill & Melinda Gates Foundation to work on research on GBS serocorrelates of protection to inform vaccine trials, and travel expenses from Pfizer to attend a meeting on an investigator-led project on GBS. S. A. M. has collaborated on GBS grants funded by GlaxoSmithKline and by Pfizer and received personal fees for being member of its advisory committee; he has also collaborated on a GBS grant funded by Minervax. L. D. V. has received a grant from ZonMW. K. M. and N. J. R. collaborated in 2016 on a project in which Chiesi Pharmaceutical partly funded and supplied melatonin. All other authors report no potential conflicts of interest. All authors have submitted the ICMJE Form for Disclosure of Potential Conflicts of Interest. Conflicts that the editors consider relevant to the content of the manuscript have been disclosed.
