# Supplementary material for: Neonatal Encephalopathy With Group B Streptococcal Disease Worldwide: Systematic Review, Investigator Group Datasets, and Meta-analysis
Source: Clin Infect Dis. 2017 Nov 6;65(Suppl 2):S173–89. doi: 10.1093/cid/cix662 (PMC5850525; doi:10.1093/cid/cix662)

# The burden of Group B *Streptococcus* worldwide for pregnant women, stillbirths and children

## Paper 9: Neonatal encephalopathy with Group B *Streptococcus* disease worldwide: systematic review, investigator group datasets, and meta-analysis

## Supplementary information

## *Contents*

**Paper 9: Neonatal encephalopathy with Group B *Streptococcus* disease worldwide: systematic review, investigator group datasets, and meta-analysis** ........ 1

**Supplementary information** ........ 1

Contents ........ 2

**Table S1:** Case definitions ........ 3

**Table S2**: Search terms ........ 4

**Table S3**: Inclusion and exclusion criteria ........ 5

**Table S4:** Clinical criteria for the definition of neonatal encephalopathy and need for therapeutic hypothermia ........ 6

**Table S5**: Outcome of contact with investigator group ........ 8

**Supplementary Figure S1**: Meta-analysis of Group B Streptococcus disease amongst infants with neonatal encephalopathy presumed to be due to hypoxia-ischemia meeting criteria for therapeutic hypothermia by IAP screening policy ........ 10

**Supplementary Figure S2**: Meta-analysis of mortality before discharge amongst infants with neonatal encephalopathy, by Group B Streptococcus disease ........ 11

*Table S1: Case definitions*

| | Definition | ICD-10 Code |
|---|---|---|
| **Neonatal encephalopathy** | A disturbance of neurological function: newborn brain dysfunction. 'a disturbance of neurological function in the earliest days of life in the term infant manifested by difficulty initiating and maintaining respiration, depression of tone or reflexes, abnormal level of consciousness, and often by seizures' (Leviton& Nelson 1992) | P91.60 |
| **Hypoxic-ischemic encephalopathy (HIE)** | Encephalopathy in the newborn in the presence of clear evidence of an intrapartum insult including acidosis on cord or early neonatal blood gases and decreased Apgar scores. | P91.60 |
| **Therapeutic hypothermia** | Total whole body cooling to a core temperature of 33.5C for 72 hours initiated within 6 hours of birth for the treatment of infants with HIE | |
| **Intrapartum-related death** | Death as a result of damage to the brain and other vital organs from events occurring around the time of birth | |
| **Birth asphyxia** | 'Failure to initiate respiration at birth' (ref WHO) | P21 |
| **Sepsis** | Clinical signs of pSBI *and* GBS-positive culture | A41.9 |
| **Meningitis** | Clinical signs of pSBI *and* [GBS-positive CSF culture *or* (GBS-positive blood culture *and* CSF pleocytosis)] | G03.9 |
| **Pneumonia** | Clinical signs (fast breathing, indrawing) *and* radiological evidence of pneumonia *and* GBS-positive blood culture | J18.9 |

*Table S2: Search terms*

| |
|---|
| ((Asphyxia Neonatorum [MeSH Terms] OR Hypoxic ischemic encephalopathy [All fields] OR Hypoxic ischemic encephalopathy [All fields] OR Perinatal asphyxia [All fields] OR Intrapartum asphyxia [All fields] OR Intrapartum asphyxia [All fields] OR Intrapartum hypoxia [All fields] OR Brain injury [All fields] OR Neonatal encephalopathy [All fields] OR Cooling) OR Therapeutic hypothermia [All fields] AND (infant or newborn or neonate) [MeSH Terms] OR infant [All fields] OR newborn [All fields] OR newborn infant [All fields] or new-born [All fields] or neonat* [All fields] )) |
| ***AND*** |
| ((Streptococcus agalactiae OR Group B streptococc* OR Streptococc* group B) OR Streptococcus agalactiae [MeSH Terms] OR (Infect* OR sepsis OR septic* OR bacter* OR blood culture OR hemoculture OR haemoculture)) |

*Table S3: Inclusion and exclusion criteria*

| | **Inclusion criteria** | **Exclusion criteria** |
|---|---|---|
| **Population** | Neonatal encephalopathy *or* Hypoxic-ischemic encephalopathy in term infants | Preterm infants (<35 weeks gestation)<br>Non representative sample |
| **Case definition** | Invasive GBS disease<br>Index case <90 days after birth | Cases not pathogen-specific |
| **Laboratory** | GBS confirmed by blood / CSF culture *or* PCR or latex agglutination *or* invasive post mortem sample | Skin colonization *or* endotracheal tube tip colonization or lung aspirate |
| **Search** | No language or date restrictions | Foreign language papers where it was not possible to obtain English translation |
| **Article type** | | Case reports |

*Table S4: Clinical criteria for the definition of neonatal encephalopathy and need for therapeutic hypothermia*

| Trial/ Score | Criteria for definition of HIE |
|---|---|
| Sarnat staging, (classifies severity) | Assigned grade 1,2,3 (mild, moderate, severe) HIE depending on findings of each of the following parameters: Muscle tone (normal, mild hypotonia/cortical thumbing, severe hypotonia), Alertness (hyperalert, lethargic, stuporose), Seizures (none, common, uncommon), Reflexes (brisk, mildly brisk, suppression), Primitive reflexes (normal, suppressed, suppression), Autonomic reflexes (sympathetic activation, parasympathetic activation, both systems suppressed), Cranial nerves (weak suck, weak/absent, absent), EEG (normal, first day low voltage then bursting pattern and multifocal electrographic seizures, deep periodic EEG with bursting pattern), Duration (<24 hours, 2-14 days, hours-weeks). |
| Thompson score for classifying NE | Score of 0-3 for the following parameters: Tone (normal, hyper, hypo, flaccid), Level of consciousness (normal hyperalert/ stare, lethargic, comatose), Fits (none, <3 per day, >2 per day), Posture (normal, fisting/ cycling, strong distal flexion, decerebrate), Moro reflex (normal, partial, absent), Grasp (normal, poor, absent), Suck (normal, poor, absent ± bites), Respiration (normal, hyperventilation, brief apnea, IPPV (apnea)), Fontanelle (normal, full and not tense, tense). Maximum Score = 22. Typically a cut-off of 5 or 6 is used to define NE. |
| Fenichel's modified criteria for classifying NE | Mild (grade 1) encephalopathy: Irritable or hyperalert, with either poor suck or an abnormality of tone. Moderate (grade 2) encephalopathy: Lethargic, with moderately abnormal tone, poor suck, and depressed Moro and grasp reflexes (seizures were often clinically evident). Severe (grade 3) encephalopathy: Comatose, with severely abnormal tone, absent suck, and brainstem malfunction including impaired respiratory drive. Modifications incorporated observations that infants with mild NE may have signs of not only decreased but increased tone, that seizure activity may not be clinically detectable and therefore cannot serve as a definitive feature in any grading system, and that the inclusion of duration in the clinical definition of a grade renders the scheme contradictory. |
| NIHCD criteria for cooling | Infants must meet all 3 criteria.<br>A: Infants ≥36 weeks gestation admitted to NICU with a diagnosis of fetal acidosis, perinatal asphyxia, neonatal depression or encephalopathy.<br>B: Umbilical cord/ arterial/ capillary blood pH <7.00 and/or base deficit ≥16 mmol/L within 60 minutes of birth, or pH 7.01-7.15/ base deficit 10-15.9 mmol/L and either an Apgar score of ≤5 at 10 minutes after birth, or assisted ventilation initiated at birth and continued for at least 10 minutes.<br>C: Encephalopathy defined as the presence of 1 or more signs in 3 of the following 6 categories: 1) level of |

| | |
|---|---|
| | consciousness (lethargy, stupor or coma), 2) spontaneous activity (decreased, absent), 3) posture (distal flexion, decerebrate), 4) tone (hypotonia, flaccid or hypertonia, rigid), 5) a) primitive reflexes (suck, weak, absent), b) Moro reflex (incomplete, flaccid) and 6) autonomic nervous system a) pupils (constricted, unequal, skew deviation or non-reactive to light), b) heart rate (bradycardia, variable heart rate), c) respiration (periodic breathing, apnea). |
| TOBY criteria for cooling | Infants must meet all 3 criteria.<br>A: Infants ≥36 weeks gestation and ≤6 hours with one of the following: Apgar score of ≤5 at 10 minutes after birth; continued need for resuscitation 10 minutes after birth; umbilical cord/ arterial/ capillary blood pH <7.00 and/or base deficit ≥16 mmol/L within 60 minutes of birth.<br>B: Moderate to severe encephalopathy consisting of altered state of consciousness (as shown by lethargy, stupor, or coma) and at least one or more of the following; hypotonia, abnormal reflexes, including oculomotor or pupillary abnormalities, an absent or weak suck, clinical seizures.<br>C: At least 30 minutes duration of aEEG recording that shows abnormal background activity or seizures (normal background with some seizure activity, moderately abnormal activity, suppressed activity, or continuous seizure activity). |
| AAP criteria for cooling | Infants must meet 2 criteria.<br>A: Umbilical cord blood or blood of pH ≤7.0 or a base deficit ≥16 mmol/L within the first hour of birth, history of an acute perinatal event, a 10-minute Apgar score <5, or assisted ventilation initiated at birth and continued for at least 10 minutes.<br>B: Moderate-severe encephalopathy on neurologic examination. If preferential head cooling is used, an abnormal background activity on EEG or aEEG is also required. |

*Table S5: Outcome of contact with investigator group*

| Country | Author | Location | Data received (Y/N) | If N, reason why data not received |
|---|---|---|---|---|
| Australia | Cheong, J | Melbourne | Y | |
| Australia | Jacobs, SE | ICE trial | Y | |
| Canada | Wintermark, P | Montreal | Y | |
| Canada | Shah, P | National | Y | |
| India | Thayyil, S | Kerala | Y | |
| India | Thayyil, S | Multi-site | Y | |
| Ireland | Hayes, B | Dublin | Y | |
| Malaysia | Boo, NY | Multi-site | Y | |
| Multi-site | Gunn, A | CoolCap trial | Y | |
| Multi-site | Azzopardi, D Edwards D | Toby Xenon trial | Y | |
| Multi-site | Shankaran, S | NICHD cooling trial | Y | |
| Nepal | Ellis, M | Kathmandu | Y | |
| Netherlands | De Vries, L | Utrecht | Y | |
| South Africa | Kali, G | Cape Town | Y | |
| Spain | Garcia-Alix, A | Barcelona | Y | |
| Turkey | Okumus.N | Ankara | Y | |
| Uganda | Tann, C | Kampala | Y | |
| UK | Gale C | National | Y | |
| UK | Tann C, Robertson NJ | London | Y | |
| UK | Thoresen, M | Bristol | Y | |
| UK/Netherlands | Cowan, F | London/Utrecht | Y | |
| USA | Glass, H | UCSF, California | Y | |
| USA | Massaro, A | Washington DC | Y | |
| USA | Walsh, B | Boston | Y | |
| USA | Jenster, M | California | Y | |
| USA | Johnson, CT | Maryland | Y | |
| Switzerland | Hagmann, C | | N | GBS data not collected |
| UK | Edwards, D Azzopardi, D | TOBY trial | N | GBS data not collected |
| Australia/New Zealand | ANZNN | National | N | Unable to provide data within time frame |
| Canada | Chau, V | | N | Unable to provide data within time frame |
| South Africa | Velaphi, S | Johannesburg | N | Unable to provide data within time frame |
| South Africa | Horn, A | | N | Unable to provide data within time frame |
| UK | Heep, A | Bristol | N | Unable to provide data within time frame |
| USA | Jenkins, D | | N | Unable to provide data within time frame |
| Australia | Shulzke, S | | N | No response |
| Austria | Simbruner, G | | N | No response |

| | | | |
|---|---|---|---|
| Brazil | Galvao, T | N | No response |
| China | Shao, X | N | No response |
| China | Cao, C | N | No response |
| China | Lin, ZL | N | No response |
| DRC | Naulaers G | N | No response |
| Egypt | Hassanein, S | N | No response |
| Greece | Xanthou, M | N | No response |
| India | Kumar, S | N | No response |
| Italy | Filippi, L | N | No response |
| Italy | Buonocore, G | N | No response |
| Italy | Celik, Y | N | No response |
| Kuwait | Elbahtiti, A | N | No response |
| Netherlands | De Haan, T | N | No response |
| Netherlands | Zonnenberg, IA | N | No response |
| USA | Angeles, D | N | No response |
| China | Sun, J | N | Email address not valid/ not successfully received |
| India | Baht, V | N | Email address not valid/ not successfully received |
| India | Memon, S | N | Email address not valid/ not successfully received |
| USA | Christensen, R | N | Email address not valid/ not successfully received |

*Supplementary Figure S1: Meta-analysis of Group B Streptococcus disease amongst infants with neonatal encephalopathy presumed to be due to hypoxia-ischemia meeting criteria for therapeutic hypothermia by IAP screening policy*

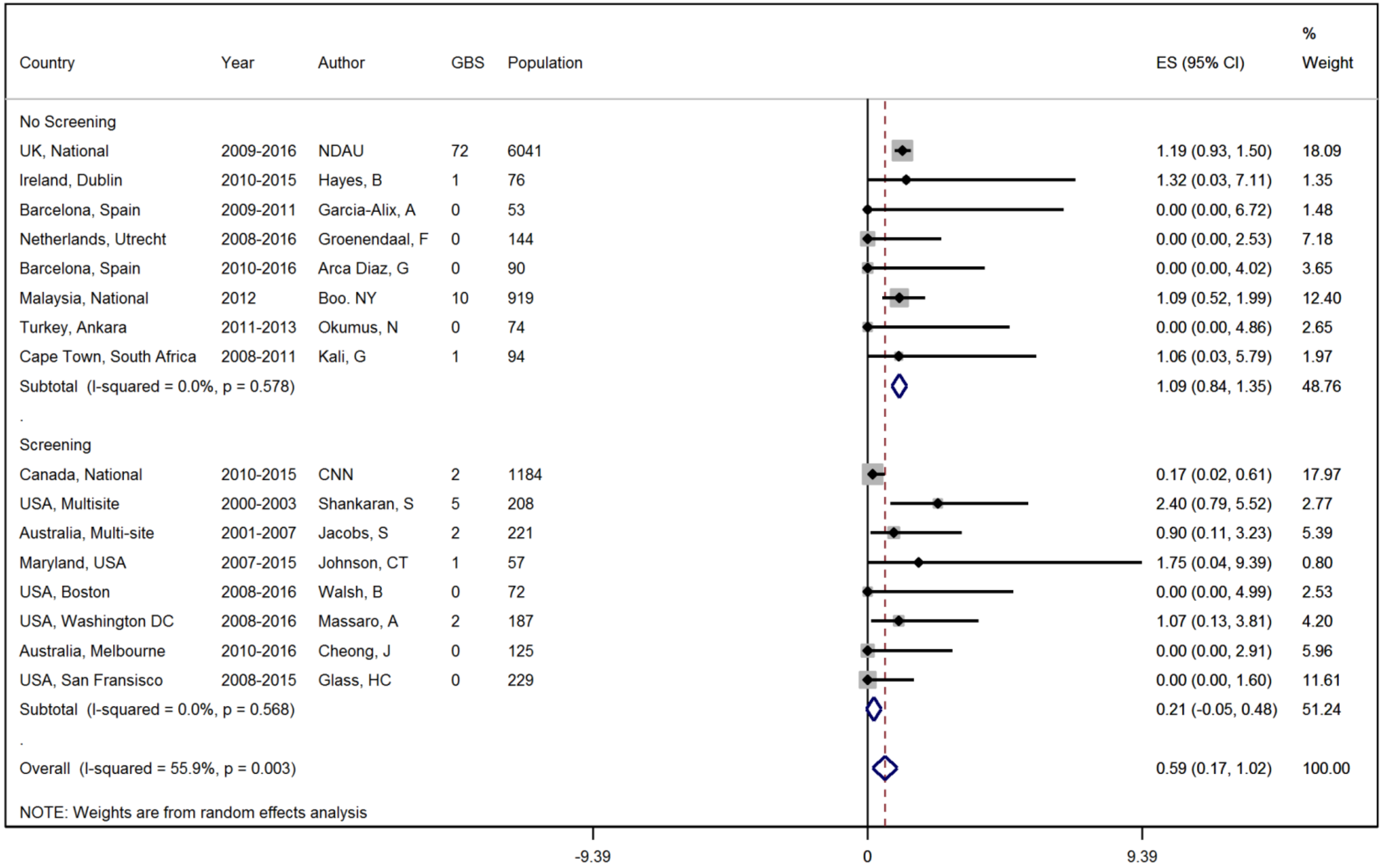

*Supplementary Figure S2: Meta-analysis of mortality before discharge amongst infants with neonatal encephalopathy, by Group B Streptococcus disease*

| Study or Subgroup | | GBS associated NE Events | GBS associated NE Total | NE without GBS Events | NE without GBS Total | Weight | Risk Ratio M-H, Random, 95% CI |
|---|---|---|---|---|---|---|---|
| Australia | Jacobs, S | 1 | 3 | 57 | 218 | 4.1% | 1.27 [0.25, 6.41] |
| Canada | CNN | 1 | 2 | 169 | 1182 | 5.4% | 3.50 [0.87, 14.08] |
| International | Gunn, A | 1 | 1 | 57 | 233 | 13.0% | 3.05 [1.33, 7.01] |
| Ireland | Hayes, B | 1 | 1 | 16 | 75 | 11.3% | 3.45 [1.39, 8.56] |
| Malaysia | Boo, NY | 4 | 10 | 140 | 909 | 14.4% | 2.60 [1.20, 5.63] |
| Netherlands | Groenendaal, F | 0 | 4 | 63 | 192 | 1.6% | 0.30 [0.02, 4.25] |
| South Africa | Kali, G | 0 | 1 | 14 | 98 | 1.9% | 1.71 [0.15, 19.72] |
| Uganda | Tann, C | 2 | 3 | 68 | 205 | 13.2% | 2.01 [0.88, 4.58] |
| UK | NDAU | 9 | 72 | 629 | 5969 | 19.9% | 1.19 [0.64, 2.20] |
| USA | Johnson, CT | 0 | 1 | 3 | 56 | 1.7% | 4.07 [0.30, 55.16] |
| USA | Massaro, A | 1 | 2 | 29 | 185 | 5.2% | 3.19 [0.77, 13.27] |
| USA | Shankaran, S | 2 | 5 | 60 | 203 | 8.3% | 1.35 [0.45, 4.04] |
| **Total (95% CI)** | | | **105** | | **9525** | **100.0%** | **2.07 [1.47, 2.91]** |
| Total events | | 22 | | 1305 | | | |

Heterogeneity: Tau$^2$ = 0.05; Chi$^2$ = 13.00, df = 11 (P = 0.29); I$^2$ = 15%

Test for overall effect: Z = 4.17 (P < 0.0001)

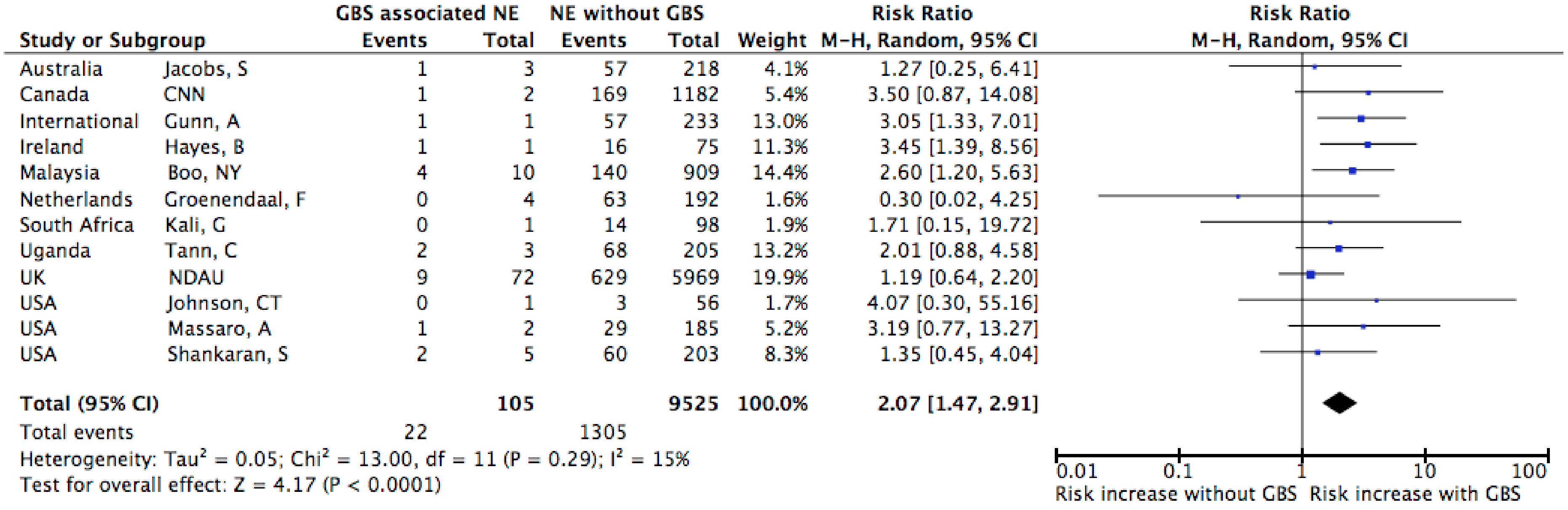

Supplement: Supplement-material [file cix662_suppl_supplement-material.pdf]
